# Prevalence of Antidepressant Prescription or Use in Patients with Acute Coronary Syndrome: A Systematic Review

**DOI:** 10.1371/journal.pone.0027671

**Published:** 2011-11-22

**Authors:** Matthew J. Czarny, Erin Arthurs, Diana-Frances Coffie, Cheri Smith, Russell J. Steele, Roy C. Ziegelstein, Brett D. Thombs

**Affiliations:** 1 Department of Medicine, Brigham and Women's Hospital, Boston, Massachusetts, United States of America; 2 Harvard Medical School, Boston, Massachusetts, United States of America; 3 Lady Davis Institute for Medical Research, Jewish General Hospital, Montreal, Quebec, Canada; 4 Department of Medicine, Rhode Island Hospital, Providence, Rhode Island, United States of America; 5 Brown Alpert Medical School, Providence, Rhode Island, United States of America; 6 Harold E. Harrison Medical Library, Johns Hopkins Bayview Medical Center, Baltimore, Maryland, United States of America; 7 Department of Mathematics and Statistics, McGill University, Montreal, Quebec, Canada; 8 Department of Psychiatry, McGill University, Montreal, Quebec, Canada; 9 Department of Epidemiology, Biostatistics, and Occupational Health, McGill University, Montreal, Quebec, Canada; 10 Department of Medicine, McGill University, Montreal, Quebec, Canada; 11 School of Nursing, McGill University, Montreal, Quebec, Canada; 12 Department of Medicine, Johns Hopkins University School of Medicine, Baltimore, Maryland, United States of America; Virginia Commonwealth University, United States of America

## Abstract

**Background and Objectives:**

Depression is common among acute coronary syndrome (ACS) patients and is associated with poor prognosis. Cardiac side effects of older antidepressants were well-known, but newer antidepressants are generally thought of as safe to use in patients with heart disease. The objective was to assess rates of antidepressant use or prescription to patients within a year of an ACS.

**Methods:**

PubMed, PsycINFO, and CINAHL databases searched through May 29, 2009; manual searching of 33 journals from May 2009 to September 2010. Articles in any language were included if they reported point or period prevalence of antidepressant use or prescription in the 12 months prior or subsequent to an ACS for ≥100 patients. Two investigators independently selected studies for inclusion/exclusion and extracted methodological characteristics and outcomes from included studies (study setting, inclusion/exclusion criteria, sample size, prevalence of antidepressant prescription/use, method of assessing antidepressant prescription/use, time period of assessment).

**Results:**

A total of 24 articles were included. The majority were from North America and Europe, and most utilized chart review or self-report to assess antidepressant use or prescription. Although there was substantial heterogeneity in results, overall, rates of antidepressant use or prescription increased from less than 5% prior to 1995 to 10–15% after 2000. In general, studies from North America reported substantially higher rates than studies from Europe, approximately 5% higher among studies that used chart or self-report data.

**Conclusions:**

Antidepressant use or prescription has increased considerably, and by 2005 approximately 10% to 15% of ACS patients were prescribed or using one of these drugs.

## Introduction

Major depressive disorder (MDD) is present in approximately 20% of patients with coronary heart disease (CHD), including acute coronary syndrome (ACS) patients [1], [2]. Depression impacts quality of life post-ACS [3], and many studies have linked MDD or depressive symptoms to poor prognosis [4].

The rate of self-reported antidepressant use increased from approximately 7% to 12% among US adults from 1996 to 2005 [5]. In Europe, just under 4% of respondents from 6 countries surveyed between 2001 and 2003 reported 12-month antidepressant use [6]. The potential for tricyclic antidepressants (TCAs) and monoamine oxidase inhibitors (MAOIs) to cause serious and potentially fatal side effects has limited their use in cardiac populations [7], [8]. On the other hand, selective serotonin reuptake inhibitors (SSRIs) have generally been accepted as safe in cardiac patients because they appear to lack these side effects [9]–[11]. Several clinical trials have examined the efficacy of SSRIs in cardiac populations [9], [10], [12]–[14] and have reported effect sizes that are similar to those from non-cardiac populations [15]–[18]. Concerns have been raised in recent years, however, about previously unrecognized adverse side effects of SSRIs potentially relevant to CHD patients [19]–[21], as well as potentially dangerous interactions between SSRIs and commonly used cardiac medications [22]–[26].

No systematic reviews have characterized the rate of antidepressant use or prescription in ACS patients, which was the objective of this review.

## Methods

Reporting of the study was based on guidelines established by the Meta-analysis of Observational Studies in Epidemiology statement [27].

### Data Sources and Searches

Potentially eligible articles were identified from the PubMed, PsycINFO, and CINAHL databases, searched on May 29, 2009. Search strategies (Supporting Information S1) were designed to identify articles that reported on ACS patients in association with depression or antidepressant use. In addition, manual searching of 33 cardiology, psychiatry, and general medicine journals (Supporting Information S2) was conducted to identify articles published subsequent to the electronic database search (May 1, 2009 to September 30, 2010).

### Study Selection

Eligible articles included published articles of original research in any language that reported point or period prevalence of antidepressant use or prescription in the 12 months prior or subsequent to an ACS, defined as unstable angina, non-ST-elevation myocardial infarction (MI), or ST-elevation MI, for at least 100 patients. Antidepressants included SSRIs, serotonin-norepinephrine reuptake inhibitors (SNRIs), TCAs, MAOIs, and atypical antidepressants. Studies with data on only a single antidepressant class were excluded. Studies with inclusion criteria that required antidepressant use or a condition associated with antidepressant use (e.g., depressive disorders) and studies that utilized antidepressants as part of an intervention were excluded unless they reported data on antidepressant prescription or use for all patients assessed for study eligibility. Non-published studies, studies published in abstract form only, letters, editorials, and case series or case reports were excluded. When multiple articles were published on the same cohort or portions of the same cohort, the article with the most complete data was included. Studies with mixed populations were included if data for ACS patients were reported separately.

Two investigators independently reviewed articles for eligibility. If either deemed an article potentially eligible based on title/abstract review, then a full-text review was completed. Disagreements after full-text review were resolved by consensus of three investigators, including the two reviewers. Non-English articles were evaluated by one investigator with the assistance of a translator. As necessary, authors were contacted to clarify information relevant to determining eligibility.

### Data Extraction

Two investigators independently extracted data from included studies into a standardized spreadsheet with discrepancies resolved via consensus. Extracted data included the study setting (North America, Europe, other), study inclusion/exclusion criteria, sample size, prevalence of antidepressant prescription/use (total and by antidepressant class), method of assessing antidepressant prescription/use, and time period of assessment. Methods of assessment were classified as administrative database, review of medical record, observation of medication containers, and patient self-report. The time period was recorded as the midpoint of the range of years when data were collected. When antidepressant use was assessed at multiple time points for the same patients, the time point closest to the index ACS was used. Authors were contacted as necessary for clarification.

One included article [28] used province-wide administrative data from Ontario, Canada to determine the percentage of post-MI patients aged 65 or older who were prescribed an antidepressant in the first 6 months post-MI. Antidepressant prescriptions were reported on a quarterly basis from the second calendar quarter of 1993 to the first calendar quarter of 2002. Numerical data were provided for the first and last quarters of the study, with scatterplot data provided for other quarters. Original data were no longer available (personal communication, Muhammad Mamdani, May 17, 2010). Therefore, two investigators estimated quarterly prevalence data from the published scatterplot, and all estimates were within 0.1% of each other. For the purposes of the present study, data are presented for one calendar quarter per year (second quarter 1993 through second quarter 2001).

### Data Synthesis and Analysis

Included studies were evaluated to determine if there was sufficient clinical and methodological similarity to support a pooled meta-regression of the rate of antidepressant prescription or use over time, factoring in setting (North America versus Europe and other) and method of assessment (administrative data versus self-report or chart review). However, excluding the study by Benazon et al. [28] that provided quarterly data over an approximately 10-year period, there were too few studies overall and not enough coverage across time, setting, and assessment method to reasonably pool all of the data from the other studies with meta-regression. Therefore, a qualitative synthesis was done for all studies in the review and a quantitative meta-analysis for a subset of studies.

Specifically, we determined that there was a sufficient number of studies to assess the association of time and setting with antidepressant prescription or use among studies that ascertained rates using non-administrative data (e.g., self-report or chart review) with the midpoint of data collection from October 1997 or later. There was not enough coverage of studies to extend the analysis prior to October 1997 or to include studies with administrative data.

For this subset of studies, we first performed an overall meta-analysis and then proceeded to fit meta-regressions controlling for region and for time. The I^2^ statistic was used to assess heterogeneity for the meta-analysis [29], and radial and funnel plots were generated to assess model assumptions. The R statistical software was used to analyze the results via the *metafor* library [30], [31]. The main meta-regression model was fit using Restricted Maximum Likelihood estimators with the rate of antidepressant usage as the outcome.

## Results

### Search Results

**Figure 1:**
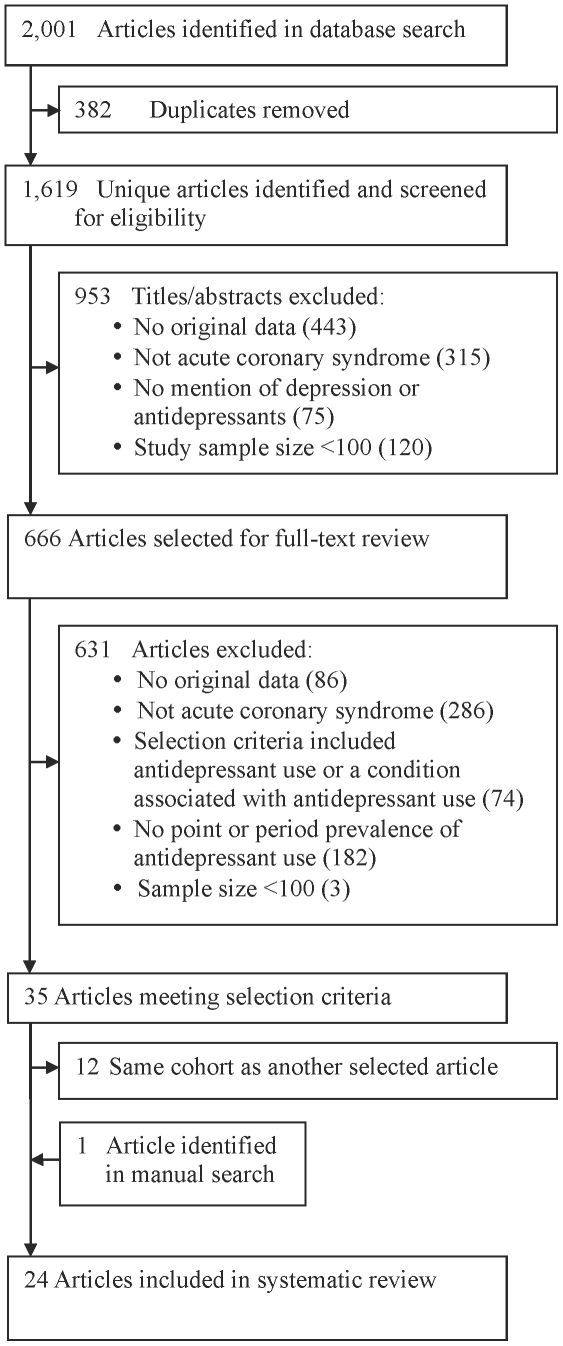
Flow Diagram of Study Selection Process.

The database search yielded 1,619 unique citations (Figure 1), including 666 selected for full text review and 35 that met inclusion criteria. Of the 35 eligible articles, 12 reported on the same cohort, leaving 23 unique articles for review. One additional eligible article was identified through manual search, resulting in a total of 24 included articles [28], [32]–[54].

### Characteristics of Included Studies

As shown in Table 1, the 24 included studies reported data on antidepressant prescription or use from April 1993 through December 2005. Data from one published study by Benazon et al. [28] reported antidepressant prescription rates in the 6 months following hospital discharge for patients surviving at least 6 months from the 2^nd^ quarter of 1993 to the 1^st^ quarter of 2002. There were 13 studies from North America, 8 from Europe, and 1 each from Japan, Australia, and New Zealand. As shown in Table 2, only 3 studies used data from administrative databases, including the Benazon et al. study [28]. One study reviewed patients' medication containers, and 20 used self-report, medical chart review, or a combination of the two. Sample sizes in the 3 studies that used administrative data ranged from 1,637 to 8,887 patients. For other studies, the range was from 126 to 2,509 patients. Of the 24 studies, 15 reported antidepressant prescription or use during the index ACS admission or at discharge, 4 reported pre-admission prescription or use, and 5 studies reported post-ACS prescription or use. One study reported antidepressant use among women aged <65 years [44], 2 studies did not report gender composition [28], [32], and the rest of the studies included predominantly males (57% to 98%).

**Table 1:** Characteristics of Studies Included in the Systematic Review.

First Author, Year	Country	Patients	N	% MI	MeanAge(Years)	% Male	% White	Years of Study
Benazon, 2005^a^	Canada	MI admission, survived ≥6 months postdischarge; ≥65 years; no MI 3 years prior to index MI	1637–2231	100%	NR	NR	NR	1993–2002
Grunau, 2006	Canada	MI admission; ≥66 years	5559	100%	NR	NR	NR	1994–1995
Lesperance, 2000	Canada	Unstable angina admission; documented CAD	430	0%	62	71%	NR	1994–1996
Parakh, 2008	USA	MI admission	284	100%	65	57%	87%	1995–1996
Sauer, 2001	USA	First MI admission; 30–65 years	653	100%	52^b^	68%^b^	NR	1995–1997
Lauzon, 2003	Canada	MI admission	464	100%	60^c^	79%^c^	96%^c^	1996–1998
Grace, 2005	Canada	ACS admission	913	53%	62	65%	NR	1997–1999
Rumsfeld, 2003	USA	ACS admission	1957	50%	65	98%	89%	1998–1999
Frasure-Smith, 2008	Canada	ACS admission; cardiac catherization	804	NR	60	81%	NR	1999–2001
Corser, 2006	USA	ACS admission	521	NR	60	64%	84%	2002–2003
Mallik, 2006	USA	MI admission	2361	100%	61	68%	NR	2003–2004
Grace, 2008	Canada	ACS admission	505	NR	62	77%	84%	2003–2004
Huffman, 2006	USA	MI admission	131	100%	62	80%	NR	2003–2005
Zimmermann-Viehoff, 2010	Sweden	Women <65 years admitted for ACS	266	36%	56	0%	NR	1991–1993
Monster, 2004	Denmark	First MI admission	8887	100%	72	62%	NR	1994–2002
Dickens, 2007	UK	MI admission	507	100%	59^d^	71%^d^	95%^d^	1997–1999
Kaptein, 2006	Netherlands	MI admission	475	100%	61	81%	NR	1997–2000
Pitzalis, 2001	Italy	MI admission <70 years	128	100%	54^e^	86%^e^	NR	1998–2000
Sorensen, 2005	Denmark	MI admission <70 years	889	NR	60	73%	NR	1999–2000
Nakatani, 2005	Japan	MI admission	2509	100%	64	77%	NR	1999–2003
Costa Dias, 2005	Portugal	ACS admission	240	71%	59	85%	NR	2001–2002
Bhattacharyya, 2007	UK	ACS admission; 18 to 90 years with paid employment	126	NR	55	88%	85%	2001–2004
Parker, 2008	Australia	ACS admission	489	65%	66	70%	NR	2001–2003
Wong, 2008	NZ	ACS admission	276	63%	64	71%	NR	2005

ACS = acute coronary syndrome; CAD = coronary artery disease; MI = myocardial infarction; NR = Not reported; NZ = New Zealand; UK = United Kingdom; USA = United States of America.

a Provincial database with quarterly data evaluated from 2^nd^ quarter of 1993 to 1^st^ quarter of 2002.

b Excluding 5 patients with “other” antidepressant use.

c Based on 550 patient enrolled in the study at index hospitalization.

d Based on 527 patients enrolled in study.

e Based on 103 patients.

**Table 2:** Antidepressant Prescription or Use in Included Studies.

First Author, Year	Country	Method of Antidepressant Use Assessment	Time of Antidepressant Use Assessment	N (%) withAntidepressant	Classes of AntidepressantsUsed
Benazon, 2005^a^	Canada	Administrative database	Within 6 months post-MI	128–357 (8% to 16%)	1993, Q2: SSRI = 29; TCA = 106. 2002, Q1: SSRI = 217; TCA = 126
Grunau, 2006	Canada	Administrative database	Within 12 months pre-MI	471 (9%)	NR
Lesperance, 2000	Canada	Chart review	Discharge	18 (4%)	NR
Parakh, 2008	USA	Chart review	Discharge	14 (5%)	NR
Sauer, 2001	USA	Self-report	Within 1 week pre-MI	18 (3%)	SSRI = 13; Other = 5
Lauzon, 2003	Canada	Self-report	12 months post-MI	13 (3%)	NR
Grace, 2005	Canada	Self-report	During admission	42 (5%)	NR
Rumsfeld, 2003	USA	Chart review	Discharge	254 (13%)	NR
Frasure-Smith, 2008	Canada	Medicine containers	2 months post-ACS	68 (8%)	NR
Corser, 2006	USA	Chart review	Discharge	61 (12%)	NR
Mallik, 2006	USA	Chart review	Discharge	231 (10%)	NR
Grace, 2008	Canada	Self-report	9 months post-ACS	48 (10%)	SSRI = 27; TCA = 6; SNRI = 8; Other = 7
Huffman, 2006	USA	Chart review	During admission	16 (12%)	NR
Zimmermann-Viehoff, 2010	Sweden	Chart review/Self-report	During admission	0 (0%)	0
Monster, 2004	Denmark	Administrative database	Within 90 days pre-MI	540 (6%)	SSRI = 289; Other = 251
Dickens, 2007	UK	Chart review	Discharge	21 (4%)	NR
Kaptein, 2006	Netherlands	Self-report	3 months post-MI	15 (3%)	NR
Pitzalis, 2001	Italy	Self-report	During admission	0 (0%)	0
Sorensen, 2005	Denmark	Chart review	Discharge	39 (4%)	NR
Nakatani, 2005	Japan	Chart review/Self-report	During admission	124 (5%)	NR
Costa Dias, 2005	Portugal	Self-report	Prior to admission	2 (1%)	NR
Bhattacharyya, 2007	UK	Chart review	During admission	8 (6%)	NR
Parker, 2008	Australia	Self-report	During admission	32 (7%)	SSRI = 17; TCA = 12; MAOI = 4
Wong, 2008	NZ	Self-report	During admission	11 (4%)	NR

MAOI = monoamine oxidase inhibitor; NR = Not reported; NZ = New Zealand; Q1 = 1^st^ quarter; Q2 = 2^nd^ quarter; SNRI = serotonin-norepinephrine reuptake inhibitor; SSRI = selective serotonin reuptake inhibitor; TCA = tricyclic antidepressant;

a Provincial database with quarterly data evaluated from 2^nd^ quarter of 1993 to 1^st^ quarter of 2002.

### Prevalence of Antidepressant Prescription or Use

**Figure 2:**
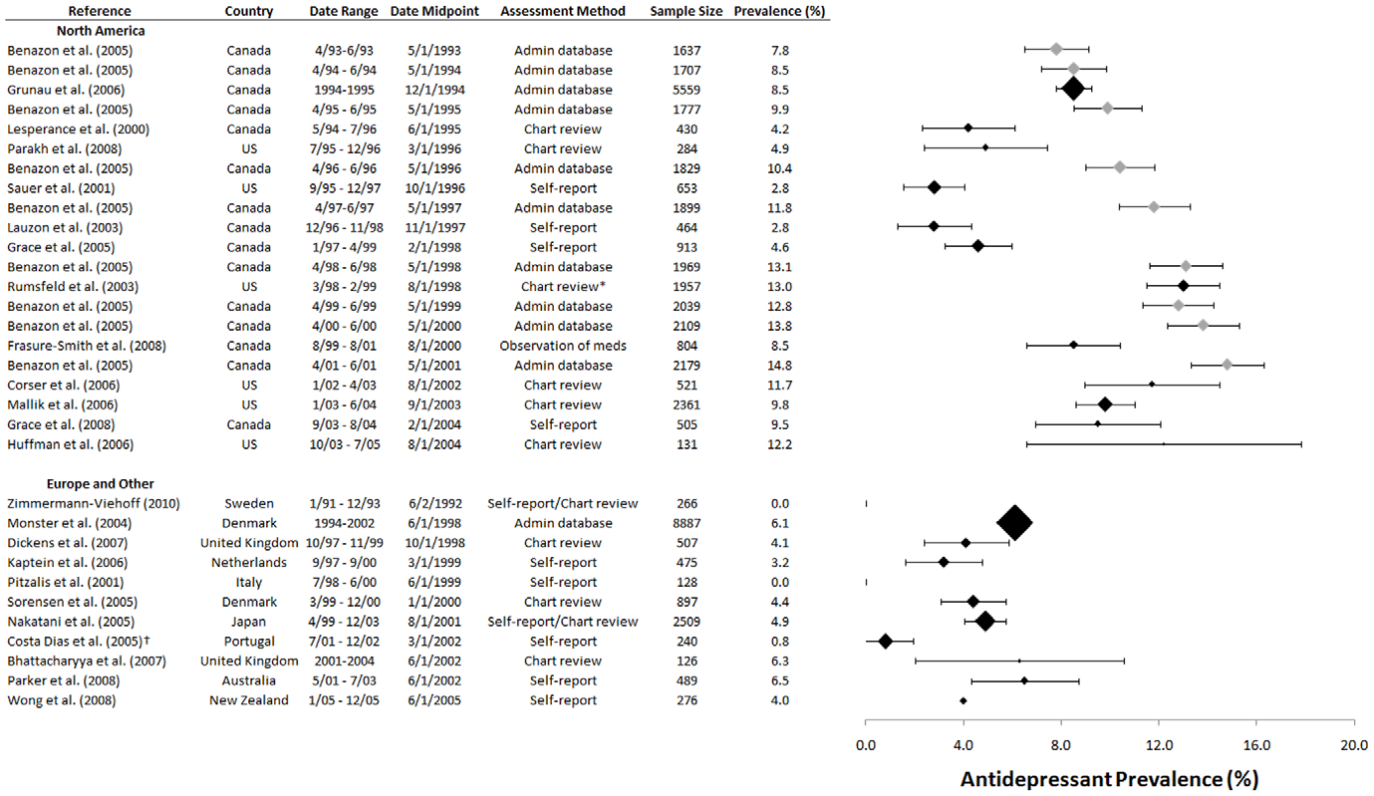
Prevalence of Antidepressant Use in ACS Patients. Studies are grouped by geographic region and sorted by midpoint of data collection period. Benazon et al. [28] rates are denoted with grey markers. Error bars are +/−1.96 standard errors, and marker sizes are weighted according to the inverse of the squared standard error. *Medication information was abstracted from patient charts but only prescriptions actually filled were included. ^†^Study reported, “only two patients were taking antidepressants prior to hospitalization,” which was coded as being assessed at hospital admission.

As shown in Figure 2, the rate of patients prescribed or using antidepressants increased from 1993 to 2005. Results from the Benazon et al. study are shown separately for 9 cohorts/years (1993 to 2001) [28]. Generally, studies from North America reported substantially higher rates than studies from Europe. Based on self-report or chart review, the only study from North America from 1995 or earlier [33] reported a rate of 4.2%, whereas the only pre-1995 European study, which reported on women under age 65 from Sweden, reported that no patients were using antidepressants upon admission for ACS [44]. One other study, which reported on Italian patients under the age of 70 admitted for MI from 1998 to 2000, similarly reported that no patients were using antidepressants at the time of admission [48]. Rates of antidepressant use or prescription among North American studies that used self-report or chart review from 2000 onward ranged from 9.5% to 13.0%, compared to 0.8% to 6.5% among non-North American studies in the same time period.

Among studies of administrative databases, rates in North America ranged from 7.8% to 9.9% from 1993 to 1995 and increased to 13.8% to 14.8% in studies from 2000 onward. The only European study that used administrative data was a Danish study that reported a rate of 6.1% of 8,887 patients with antidepressant prescriptions in the years 1994 to 2002 [45]. During approximately the same period, rates reported by Benazon et al. from Ontario, Canada increased from 7.8% in the second quarter of 1993 to 15.7% by the first quarter of 2002 (Benazon data in Figure 2 through the second quarter of 2001) [28].

Only five studies reported rates of use or prescription by antidepressant class 28,35,42,45,53], so analysis of rates by class was not possible. However, in the study by Benazon et al. [28] the percentage of patients prescribed an SSRI increased five-fold from approximately 2% in the second quarter of 1993 to approximately 10% in the first quarter of 2002. TCA prescription, on the other hand, remained stable at approximately 6% across the study period.

**Figure 3:**
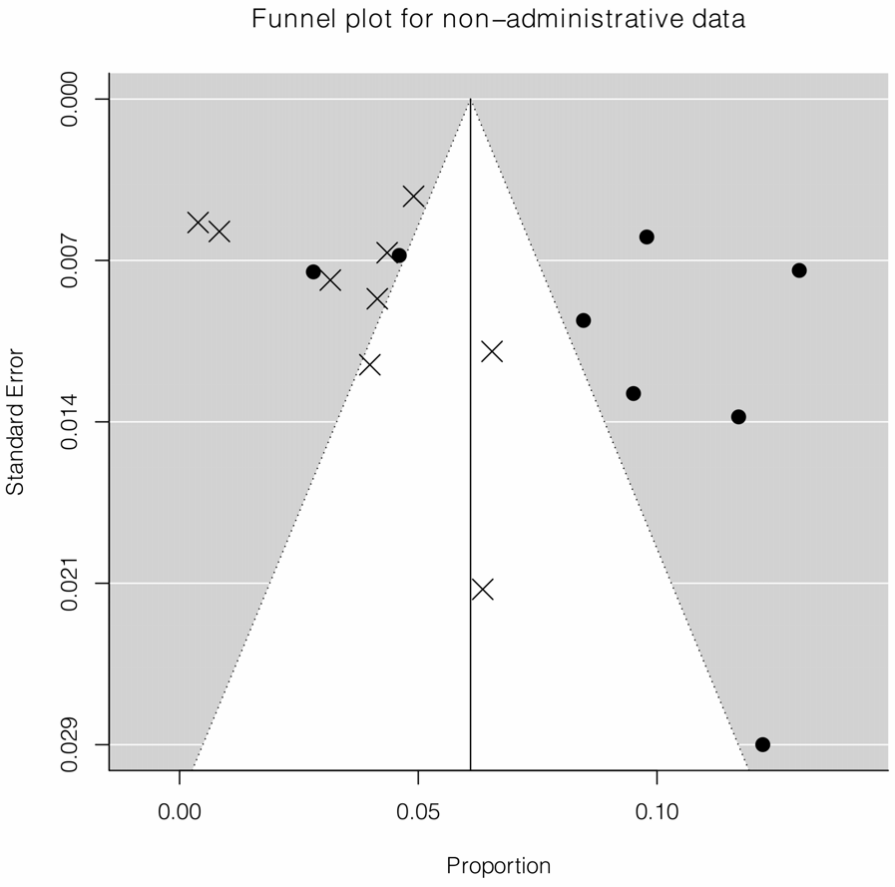
Heterogeneity of Unadjusted Prevalence Estimates. The funnel plot shows individual study prevalence estimates (x-axis) plotted against estimated standard errors (y-axis). The white cone inside the gray region indicates a 95% probability region that points would be expected to fall into assuming no heterogeneity and no publication bias. The North American studies are indicated by the filled circles and the non-North American studies are indicated by the × symbols.

**Figure 4:**
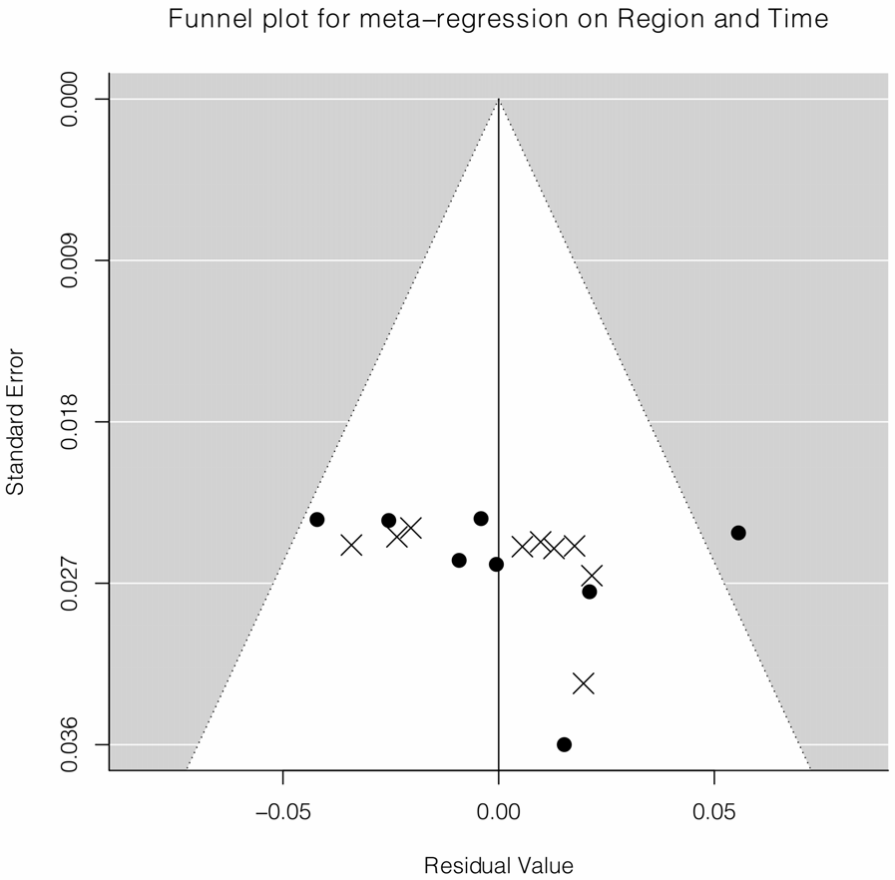
Heterogeneity of Adjusted Prevalence Estimates. The funnel plot shows the residuals from the meta-regression on setting and time (x-axis) plotted against estimated standard errors (y-axis). The white cone inside the gray region indicates a 95% probability region that points would be expected to fall into assuming the assumptions of the meta-regression were valid. The North American studies are indicated by the filled circles and the other studies are indicated by the × symbols.

There were 17 studies that assessed prevalence of antidepressant prescription or use using non-administrative databases with study midpoints of October 1997 onward and were included in the meta-analysis, including 8 studies from North America [36]–[43] and 9 studies from Europe [46]–[49], [51], [52], Japan [50], Australia [53], or New Zealand [54]. Overall, the random effects model estimate for antidepressant use for all 17 studies was 6.1% (95% confidence interval (CI) 4.3% to 7.9%). However, the I^2^ statistic was extremely large (95.7%), indicating a substantial amount of between study heterogeneity, which was also seen in the funnel plot (Figure 3). Meta-regression was done with setting (North American versus Europe and other) and time (year of midpoint of data collection) as predictor variables. The estimated difference between rate of use in North America versus Europe and other was 5.1% (95% CI 2.5% to 7.8%, p<0.001). In addition, the rate of use increased by approximately 0.5% per year (95% CI 0.0% to 1.1%, p = 0.065). Adjusting for setting and time accounted for 50% of heterogeneity in prevalence between studies from the overall meta-analysis, and the funnel plot reflected less structured heterogeneity and better model fit (Figure 4). Results did not change if transformations of the raw proportions (arcsin and log) were modeled.

## Discussion

Consistent with general population trends [5], [6], [55] the prescription or use of antidepressants has risen dramatically among ACS patients since the early 1990s. Although there was substantial heterogeneity in results, overall, rates of antidepressant prescription or use increased from less than 5% prior to 1995 to 10–15% after 2000. One study that tracked antidepressant prescribing to post-MI patients using province-wide administrative data from Ontario, Canada found that the percentage of patients with an antidepressant prescription in the 6 months following hospital discharge increased from 7.8% in 1993 to 15.7% by 2002 [28]. In the meta-regression, there was an increase of approximately 0.5% per year in studies that used non-administrative data between 1997 and 2005. As with general population data, the rate of antidepressant prescription or self-reported use was notably higher in North America than in other parts of the world, primarily Europe, with an estimated difference of approximately 5.1% among studies from 1997 onward that used self-report or chart data to establish usage rates.

There has been a striking increase in the use of antidepressant medications in the general population in the past two decades, due in large part to a significant increase in the prescribing of antidepressant medications by nonpsychiatrists [55]. One reason for this trend is likely to be the perception that antidepressants are safe to use even in patients with medical comorbidities, including heart disease [56]. Based on the results of several studies [9], [10], [12] including post hoc analysis of the Enhancing Recovery in Coronary Heart Disease Patients (ENRICHD) study [57], it has been concluded that SSRIs are safe to use in patients with heart disease [58]. Consistent with this, a recent study of antidepressant use in primary care notes “…the preponderance of evidence suggests that it is not necessary to be overly cautious when prescribing for patients who have medical comorbidities. Most studies have demonstrated that SSRIs are safe and effective in persons who have both CAD (coronary artery disease) and stroke” [56]. The side effects of the older antidepressants, however, limited their use in heart disease. TCAs can increase heart rate, produce orthostatic hypotension, decrease heart rate variability, prolong cardiac conduction, and trigger potentially lethal ventricular arrhythmias [9]. MAOIs can also produce orthostatic hypotension, but their potential to cause hypertensive crisis in patients who ingest tyramine-containing food or certain other drugs is the most concerning of their cardiovascular side effects [59].

The increased use of antidepressant drugs in ACS patients in recent years may not only reflect greater comfort with these agents, but also a greater awareness of the burden of depression in patients with CHD [2], [4]. Over the years, many informal calls have been made for increased depression treatment in heart disease patients, and recently an AHA Science Advisory recommended routine depression screening for all heart patients in order to increase the treatment rate [58]. Indeed, the problem of under-diagnosed and under-treated depression has been recognized as an important public health issue outside of cardiovascular care settings, and increasing depression treatment, including antidepressant prescribing, has been prioritized [60].

Whether depression in ACS patients is under-treated despite the more widespread prescription or use of antidepressants documented in this study is not known. Distinguishing under-use of antidepressants from appropriate use or overuse is beyond the scope of this systematic review. Nonetheless, it should be noted that overuse occurs when the risk of potential harm of using a drug outweighs the likely benefits for some patients. The overuse of antidepressants among populations who are unlikely to benefit meaningfully, is well-documented [61]. In the context of ACS, it should be pointed out that as antidepressant use increases, the potential for harm, even if small, also increases. Although the SSRIs appear to have few direct adverse cardiovascular effects, clinically important drug-drug interactions may be encountered with more widespread use in ACS patients since many of these drugs inhibit hepatic cytochrome P450 isoenzymes [62], which are involved in the metabolism of many cardiac drugs. The safety of SNRIs in ACS patients has not been as well-studied, which is important to consider since these drugs can increase blood pressure and heart rate [63]. It should be noted that recent reports raise persistent concern about potential adverse cardiovascular effects of antidepressant drugs [20], [21], [25], [26], [64]–[66], suggesting that additional studies that evaluate cardiovascular side effects of antidepressant drugs in greater numbers of patients followed for longer time periods may be warranted.

There are limitations that should be considered in interpreting the results of this study. First, although the search that was conducted was broad and resulted in the review of more than 600 full-texts of articles, it is possible that some studies that reported on the rate of antidepressant use or prescription may not have been identified. Second, there are limitations in the methods used by original studies to ascertain the rate of antidepressant prescription or use. It is well-known, for instance, that self-report methods, which were used in a number of the studies we reviewed, tend to underestimate rates of prescription and use [67]–[69].

There were not enough studies from North America versus Europe and that used administrative data versus self-report or chart data across the time frame of the studies we reviewed to synthesize all data quantitatively. Thus, we were only able to estimate the prevalence quantitatively using non-administrative database studies that were conducted from 1997 onward. With only 17 studies included in the meta-analysis and limited information on some of these, we were not able to adjust for study characteristics beyond setting and time, such as patient gender or age. Even after adjusting for these variables, there was a substantial amount of heterogeneity between studies. Thus, the meta-regression results should be interpreted with caution.

Additionally, very few studies provided information on the classes of antidepressants prescribed, which did not permit specific analysis. Furthermore, no studies with data on antidepressant prescription or use since 2005 were available, so it is not known whether or not prescription rates have continued to increase beyond the rates reported in the studies reviewed. Finally, the protocol for this review was not registered prior to initiating work on the review.

In summary, the results of this systematic review showed that the rate of antidepressant prescriptions and use has risen dramatically in recent years among ACS patients and, as in the general population, tends to be higher in North America than in other parts of the world. The fact that by 2005 between 10% and 15% of ACS patients were prescribed or using an antidepressant suggests that more patients are receiving treatment for depression, which is clearly an important problem in patients with heart disease. Alternatively, this prevalence of antidepressant use raises the possibility that antidepressants are being used by some ACS patients who may not benefit meaningfully despite being exposed to the possibility of adverse effects of the drugs themselves or to clinically significant drug-drug interactions. Careful consideration needs to be given to the balance between potential benefits and harms in prescribing antidepressant medications to patients with heart disease.

## Supporting Information

Supporting Information S1 **Search Strategies.**
(DOC)

Supporting Information S2 **Journals Included in Manual Searches.**
(DOC)
