# Supplementary material for: Prevalence of Antidepressant Prescription or Use in Patients with Acute Coronary Syndrome: A Systematic Review
Source: PLoS One. 2011 Nov 22;6(11):e27671. doi: 10.1371/journal.pone.0027671 (PMC3222644; doi:10.1371/journal.pone.0027671)
Supplement: Supporting Information S1 — Search Strategies. (DOC) [file pone.0027671.s001.doc]

**SUPPORTING INFORMATION 1: SEARCH STRATEGIES**

**CINAHL:**

S1: (MH "Coronary Disease") or (MH "Myocardial Ischemia+”)

Search mode: Find all my search terms

S2: (MH "Antidepressive Agents+") or (MH "Depression+")

Search mode: Find all my search terms

S3: S1 and S2

Search mode: Boolean/Phrase

S4: S1 and S2

Search mode: Boolean/Phrase

Limiters: Publication Type: Abstract, Accreditation, Advice and Referral Website, Anecdote, Bibliography, Biography, Book, Book Chapter, Book Review, Brief Item, Care Plan, Cartoon, Case Study, CEU, Chat Groups, Classification Term, Code of Ethics, Commentary, Commercial Website, Computer Program, Consumer/Patient Teaching Materials, Directories, Editorial, Evidence-Based Care Sheet, Exam Questions, Games, Glossary, Historical Material, Individual Testimonial Website, Information Website, Interview, Legal Cases, Letter, Listservs, Nurse Practice Acts, Nursing Diagnoses, Nursing Interventions, Obituary, Pamphlet, Pamphlet Chapter, Poetry, Proceedings, Questions and Answers, Quick Lesson, Response, Search Strategy, Software, Standards, Teaching Materials, Tracings, Website

S5: S3 not S4

Search mode: Boolean/Phrase

**PsycInfo (Boolean/Phrase search):**

S1: “myocardial ischemia”

OR “myocardial ischaemia”

OR “myocardial ischemias”

OR “myocardial ischaemias”

OR “Ischemic Heart Disease”

OR “Ischemic Heart Disesases

S2: “acute coronary syndrome”

OR “acute coronary syndromes”

OR acs

OR stemi

OR nstemi

S3: DE “Myocardial Infarctions”

S4: DE “Angina Pectoris”

AND unstable

S5: S1 or S2 or S3 or S4

S6: DE "Antidepressant Drugs"

OR DE "Bupropion"

OR DE "Citalopram"

OR DE "Fluoxetine"

OR DE "Fluvoxamine"

OR DE "Iproniazid"

OR DE "Isocarboxazid"

OR DE "Lithium Carbonate"

OR DE "Methylphenidate"

OR DE "Mianserin"

OR DE"Moclobemide"

OR DE "Molindone"

OR DE "Nefazodone"

OR DE "Nialamide"

OR DE "Nomifensine"

OR DE "Paroxetine"

OR DE "Phenelzine"

OR DE "Pheniprazine"

OR DE "Pipradrol"

OR DE "Serotonin Norepinephrine Reuptake Inhibitors"

OR DE "Sertraline"

OR DE "Sulpiride"

OR DE "Tranylcypromine"

OR DE "Trazodone"

OR DE "Tricyclic Antidepressant Drugs"

OR DE "Venlafaxine"

OR DE "Zimeldine"

S7: DE "Depression (Emotion)"

OR DE "Major Depression"

OR DE "Anaclitic Depression"

OR DE "Dysthymic Disorder"

OR DE "Endogenous Depression"

OR DE "Postpartum Depression"

OR DE "Reactive Depression"

OR DE "Recurrent Depression"

OR DE "Treatment Resistant Depression"

S8: S6 or S7

S9: S5 and S8

**PubMed:**

"Myocardial Ischemia"[Mesh] AND ("Antidepressive Agents"[MeSH] OR "Serotonin Uptake Inhibitors"[MeSH] OR "Monoamine Oxidase Inhibitors"[MeSH] OR "Depression"[Majr] OR "Depressive Disorder"[Majr]) NOT (Letter[ptyp] OR Meta-Analysis[ptyp] OR Review[ptyp] OR Editorial[ptyp] OR Practice Guideline[ptyp] OR Case Reports[ptyp] OR Comment[ptyp])
