# Supplementary material for: Prevalence of Antidepressant Prescription or Use in Patients with Acute Coronary Syndrome: A Systematic Review
Source: PLoS One. 2011 Nov 22;6(11):e27671. doi: 10.1371/journal.pone.0027671 (PMC3222644; doi:10.1371/journal.pone.0027671)
Supplement: Supporting Information S2 — Journals Included in Manual Searches. (DOC) [file pone.0027671.s002.doc]

**SUPPORTING INFORMATION 2: Journals included in manual searches**

American Heart Journal
American Journal of Cardiology
American Journal of Medicine
American Journal of Geriatric Psychiatry
American Journal of Psychiatry
Annals of Behavioral Medicine
Annals of Internal Medicine
Archives of General Psychiatry
Archives of Internal Medicine
Biological Psychiatry

British Medical Journal
Canadian Journal of Psychiatry
Canadian Medical Association Journal
Circulation
European Heart Journal
European Cardiovascular Prevention & Rehabilitation
European Journal of Heart Failure
General Hospital Psychiatry
Health Psychology
Heart
Herz

Journal of the American Medical Association
Journal of Affective Disorders
Journal of Behavioral Medicine
Journal of Cardiopulmonary Rehabilitation
Journal of the American College of Cardiology
Journal of General Internal Medicine
Journal of Psychosomatic Research
Lancet

New England Journal of Medicine
Psychosomatic Medicine
Psychosomatics
Psychotherapy and Psychosomatics
